# Comprehensive Analysis of Genes Associated With Sudden Infant Death Syndrome

**DOI:** 10.3389/fped.2021.742225

**Published:** 2021-10-15

**Authors:** Riffat Mehboob, Maher Kurdi, Mursleen Ahmad, Syed Amir Gilani, Sidra Khalid, Hisham Nasief, Abeer Mirdad, Husam Malibary, Sahar Hakamy, Amber Hassan, Meshari Alaifan, Ahmed Bamaga, Syed Adnan Shahzad

**Affiliations:** ^1^Research Unit, Faculty of Allied Health Sciences, The University of Lahore, Lahore, Pakistan; ^2^Lahore Medical Research Center, LLP, Lahore, Pakistan; ^3^Department of Pathology, Faculty of Medicine in Rabigh, King Abdulaziz University, Jeddah, Saudi Arabia; ^4^Department of Medicine, Sahiwal Medical College, Sahiwal, Pakistan; ^5^Department of Obstetric and Gynecology, Faculty of Medicine, King Abdulaziz University and Hospital, Jeddah, Saudi Arabia; ^6^Pediatric Department, East Jeddah Hospital, Jeddah, Saudi Arabia; ^7^Department of Internal Medicine, Faculty of Medicine, King Abdul Aziz University, Jeddah, Saudi Arabia; ^8^Center of Excellence in Genomic Research, King Abdulaziz University, Jeddah, Saudi Arabia; ^9^Department of Paediatrics, Faculty of Medicine, King Abdulaziz University, Jeddah, Saudi Arabia; ^10^Paediatric Department, King Faisal Specialist Hospital and Research Center, Jeddah, Saudi Arabia; ^11^Neurology and Pediatric Department, Faculty of Medicine, King Abdulaziz University Hospital, King Abdulaziz University, Jeddah, Saudi Arabia; ^12^Faculty of Medicine and University Hospital of Cologne, Institute of Virology, University of Cologne, Cologne, Germany

**Keywords:** sudden infant death syndrome, channelopathies, sodium channels, cardiac channel genes, breathing control, neuropathology

## Abstract

**Background:** Sudden infant death syndrome (SIDS) is a tragic incident which remains a mystery even after post-mortem investigation and thorough researches.

**Methods:** This comprehensive review is based on the genes reported in the molecular autopsy studies conducted on SIDS so far. A total of 20 original studies and 7 case reports were identified and included in this analysis. The genes identified in children or adults were not included. Most of the genes reported in these studies belonged to cardiac channel and cardiomyopathy. Cardiac channel genes in SIDS were scrutinized for further analysis.

**Results:** After screening and removing the duplicates, 42 unique genes were extracted. When the location of these genes was assessed, it was observed that most of these belonged to Chromosomes 11, 1 and 3 in sequential manner. The pathway analysis shows that these genes are involved in the regulation of heart rate, action potential, cardiac muscle cell contraction and heart contraction. The protein-protein interaction network was also very big and highly interactive. SCN5A, CAV3, ALG10B, AKAP9 and many more were mainly found in these cases and were regulated by many transcription factors such as MYOG C2C1 and CBX3 HCT11. Micro RNA, “hsa-miR-133a-3p” was found to be prevalent in the targeted genes.

**Conclusions:** Molecular and computational approaches are a step forward toward exploration of these sad demises. It is so far a new arena but seems promising to dig out the genetic cause of SIDS in the years to come.

## Introduction

Sudden Infant Death Syndrome (SIDS) is described as a diagnosis of exclusion and is defined as unanticipated passing away of an infant < 1 year of age that remains unsolved even after thorough inquiries that includes a review of the clinical history, performance of complete autopsy, and examination of the death scene (1). Matturri et al. (2) proposed the definitions of SIDS as “the sudden death of an infant under 1 year of age which remains unexplained after a thorough case investigation, including the performance of a complete autopsy, examination of the death scene, and review of the clinical history” should be modified by adding, at the end, the following: “a complete autopsy with an in-depth histopathologic analysis of the cardiorespiratory innervation and specialized myocardium, performed only by an experienced, reliable pathologist.” In 2004, Krous et al. defined SIDS as “The sudden unexpected death of an infant less than 1 year of age, with onset of the fatal episode apparently occurring during sleep, that remains unexplained after a thorough investigation, including performance of a complete autopsy and review of the circumstances of death and the clinical history.” This definition was elaborated by Ottaviani as, “the findings in the cardiac conduction and autonomic nervous systems detected in SIDS can be morphological substrates for the sudden unexpected death (3). In 2016, Goldstein et al. (4) reported that “there is no consensus on the use of the term SIDS, as external factors, such as prone sleep position or bed sharing, may at times explain the cause of death as positional asphyxia or accidental suffocation.”

Therefore, an in-depth histopathological analysis of the cardiac conduction system and autonomic nervous system by expert pathologists is mandatory (3). According to the American Heart Association (5), congenital heart diseases occur in 0.8% of full term live births. Anomalies in the cardiac conduction system and cardiac channelopathies should be considered to be included in congenital heart disease (6). SIDS is diagnosed by eliminating all other known illnesses. Incidence of SIDS spikes between 2 and 4 months of age and greater than 90% happens in the period of first 6 months of the life of infants. Due to the global campaign of “Back To sleep” along with other campaigns, 50-90% drop in the cases of SIDS has been recorded since 1990 (7).

SIDS and Sudden intrauterine death (SIUD) have multifactorial etiology comprising of congenital anomalies of the cardiac conduction system and the autonomic nervous system, respiratory rhythm generation and arousal from sleep (8). Based on these considerations, the new common definition for the SIDS-SIUDS complex is “The sudden death of a fetus after the 25th gestational week or infant under 1 year of age which is unexpected by history and remains unexplained after a thorough case investigation, including examination of the death scene, performance of a general autopsy and examination of the fetal adnexa” (3). Routine autopsy fails to disclose any cause of death in such cases and a more in depth histopathological analysis of the cardiac conduction system and autonomic nervous system is required. Molecular autopsy should also be followed in this regard.

Triple risk model for SIDS as described by Filiano and Kinney, suggests three factors: a vulnerable infant, a critical developmental period in homeostatic control, and an exogenous stressor(s) (9). All three factors, when present together, may lead to SIDS. Neuropathological and Cardiovascular conduction system abnormalities are the main culprits involved in the vulnerability of the infant or fetus for Sudden perinatal death when it combines with the genetic predisposition (3). Sudden cardiac death (SCD) is defined as the unexpected death without an obvious non-cardiac cause that occurs within 1 h of witnessed symptom or within 24 h of unwitnessed symptom onset (10). SCD occurs by at least these three mechanisms: Ischemia induced by a perturbation in a coronary artery with rapid development of ventricular fibrillation, arrhythmia occuring in an early-stage of acute myocardial infarction associated with coronary thrombosis, primary ventricular arrhythmia not associated with new onset ischemia, related to a cardiomyopathy or channelopathy (11).

Genetic predisposition is responsible for vulnerability of an infant toward SIDS. So, genetics does not play its role alone, but it interacts with the epidemiological factors and stressors acting in a critical developmental phase of the infant (12). It is important to understand the genetics associated with SIDS in order to prevent such casualties by taking care of modifiable risk factors in vulnerable infants. So far there is a scarcity of literature available due to limited research on this aspect which led us to write a comprehensive and updated review. Genetics play its role either directly such as in cases with medium-chain acyl-coenzyme A dehydrogenase deficiency and cardiac arrest due to long QT syndrome or indirectly by acting as a predisposing factor for SIDS (13, 14). Inheritance pattern of genes contributing to SIDS is polygenic and acts in combination with environmental risk factors, e.g., male gender, prone sleeping position, carelessness of parents, winter, seasonal variations, night, botulism etc. Immune system, cardio-respiratory function and neurophysiology related genes have been prominent so far in association with SIDS (13).

Inflammation and infections are also contributing factors in SIDS (15). Viral infections, particularly of the respiratory tract, are also among one of the etiological factors for SIDS. Epidemiology and pathology related to SIDS, provides evidence for this concept. But the results are conflicting regarding the isolation of specific viruses due to technical issues, lack of proper controls and many other reasons. However, another very important contributing factor for SIDS is an immunological response (16). Mucosal immune system is activated in SIDS in different studies (17, 18). Higher IgG and IgA immunocyte density in the tonsils of SIDS victims has been observed (18). Elevated expression of CD45+ stromal leucocytes and highly variable expression of human leukocyte antigen-antigen D(HLA-DR) and enhanced expression of HLA class I and II have also been observed in salivary glands in SIDS. It shows that the immune system is stimulated in SIDS with the release of cytokines, which then up-regulate the epithelial expression of HLA-DR (19). Bacteriological findings have also been reported and *Staphylococcal aureus* and staphylococcal endotoxins have been found to be prevalent in SIDS (20).

Altered immunological homeostasis has been hypothesized as an important factor for SIDS etiology. Cytokines are essential mediators for infant health by monitoring the cell activity during inflammation. The pro-inflammatory cytokines such as IL-1α, IL-1β, IL-6, IL-8, IL-12, IL-18, TNF-α, and IFN-γ potentiates inflammation. The anti-inflammatory cytokines, e.g., interleukin-1 receptor antagonist (IL-1ra), IL-4, IL-10, IL-11, and IL-13 control them. Cytokine genes and their variants may explain the vulnerability of infants to infection. The genes encoding IL-1, IL-6, IL-10, and TNF-α are the most studied in SIDS. Genes involved in the immune system are important in SIDS and specific haplotypes in the IL-10 gene promoter have been associated with SIDS due to infection (21). In toddlers who had SIDS, polymorphism inside the gene IL10 (inflammatory cytokine interleukin 10) at promoter place could impel the reduction in production of antibodies or it can act as a substitute to enhance production of inflammatory cytokine (22). Immunogenetics and cytokine network are very complex to understand and need further exploration (23).

According to certain studies, genetic anomalies that are associated with channelopathies (arrhythmia) may also play a part in SIDS. Main lethal arrhythmias give rise to sudden cardiac death due to genetic variations such as: CPVT (catecholaminergic polymorphic ventricular tachycardia), LQTS (long QT syndrome), SQTS (short QT syndrome) and BrS (Brugada syndrome) (24). So far about 30 genes have been found linked with that of major principal cardiac arrhythmic ailment but mainly 4 genes (RyR2, SCN5A, KCNQ1, and KCNH2) have been taken into account. The chief answerable for each ailment: SQTS (KCNH2 20% and KCNQ1 30%), CPVT (RyR2 50%), BrS (SCN5A 25%), and LQTS (SCN5A 15%, KCNH2 30% and KCNQ1 40%) (25).

Ten to fifteen percent of the SIDS cases are thought to be caused by cardiac arrhythmias (26), particularly CPVT and LQT (27, 28). Furthermore, polymorphism in genes that linked to sleep trouble disorders have been seen to be effective as a potential hazard in terms of genes for SIDS (29). Phenomenon of polymorphism is also acknowledged in 5-HTT (serotonin transporter gene) that will enhance activity of serotonin transporter, on the contrary lessening the concentrations of serotonin at endings of nerves (30, 31). In addition, polymorphism was recognized in genes that affected the enhancement of autonomic apprehensive device (EN1, TLX3, ECE1, RET, PHOX2a) (32).

The progress of SIDS lingers to be solved, but 1 out of 10 cases is linked with mutations of cardiac ion channel gene, it also include the genes that encode the βeta subunits (33). p.S206L in SCN4B and p.V54G and p.V36M mutations in SCN3B have been associated to SIDS (34). Significantly, p.V36M mutation in SCN3B has also been associated to ventricular fibrillation of unknown cause, which is a possible lethal cardiac arrhythmia, and p.S206L in SCN4B also give rise to unusual excitableness in ventricular muscle cells of rat (34, 35). On the other hand there has been one occurrence of SIDS in a baby with a p.R214Q in β1B, which is also linked with Brugada Syndrome (36). β1B regulates function of Nav1.5, significantly assigning a causal method for cardiac malfunction associated with SCN1B (37). Up till now, no variations in SCN2B have been associated to SIDS. A huge figure of genes are thought to be the genetic reason of SIDS. Regardless of innovative progress in the turf of medical science, up to this time a very few facts with regard to genetics of SIDS are present (38). Similar to other heart ailments, variation in ion channel gene is as well a potential cause for SIDS (39).

Furthermore, variations in a number of heart channel genes have also been taken into consideration as a reason of SIDS and variations in genes associated with ion channels of heart (KCNH2, LQTS,SCN5 and KCNQ) in 9.5 % of SIDS cases (40, 41). In a study, 38 out of 47 victims of SIDS were examined for alternatives in KCNH2, SCN5A, RYR2, and KCNQ1, exhibited that 8 (17%) of the victims of SIDS had variations in gene disturbing role of ion channels. Mutations in these genes will give rise to disorders of malfunctioning of ion channels which can lead to arrhythmias and sudden death due to hereditary heart ailments (42).

This study was planned to review the previous literature regarding the genetic predisposition in SIDS and investigate a link between genetic and epidemiological factors associated with SIDS.

## Methods

This is an *in silico* study based on the existing data reported in literature related to the genes associated with SIDS. Very few studies have been found and the genes identified in these studies were gathered and analyzed for various properties through bioinformatics tools. These studies included 20 original articles and 7 case reports. After deleting the repetitive genes, a total of 42 unique genes were identified which were explored further. We have analyzed these genes for computational properties, pathway analysis, gene enrichment analysis, histone methylation and other properties.

We employed ShinyGo (https://academic.oup.com/bioinformatics/article/36/8/2628/5688742), for enrichment, pathway and gene characterization. All analysis were performed on ShinyGo V0.61. For histone mark enrichment, mircoRNAs and transcription factors targets we used an integrative web enrichment (https://www.ncbi.nlm.nih.gov/pmc/articles/PMC3637064/). Protein-protein association network analysis were performed using String database.

## Results

### Genomic Architecture of Genes Involved in SIDS

**Figure 1:**
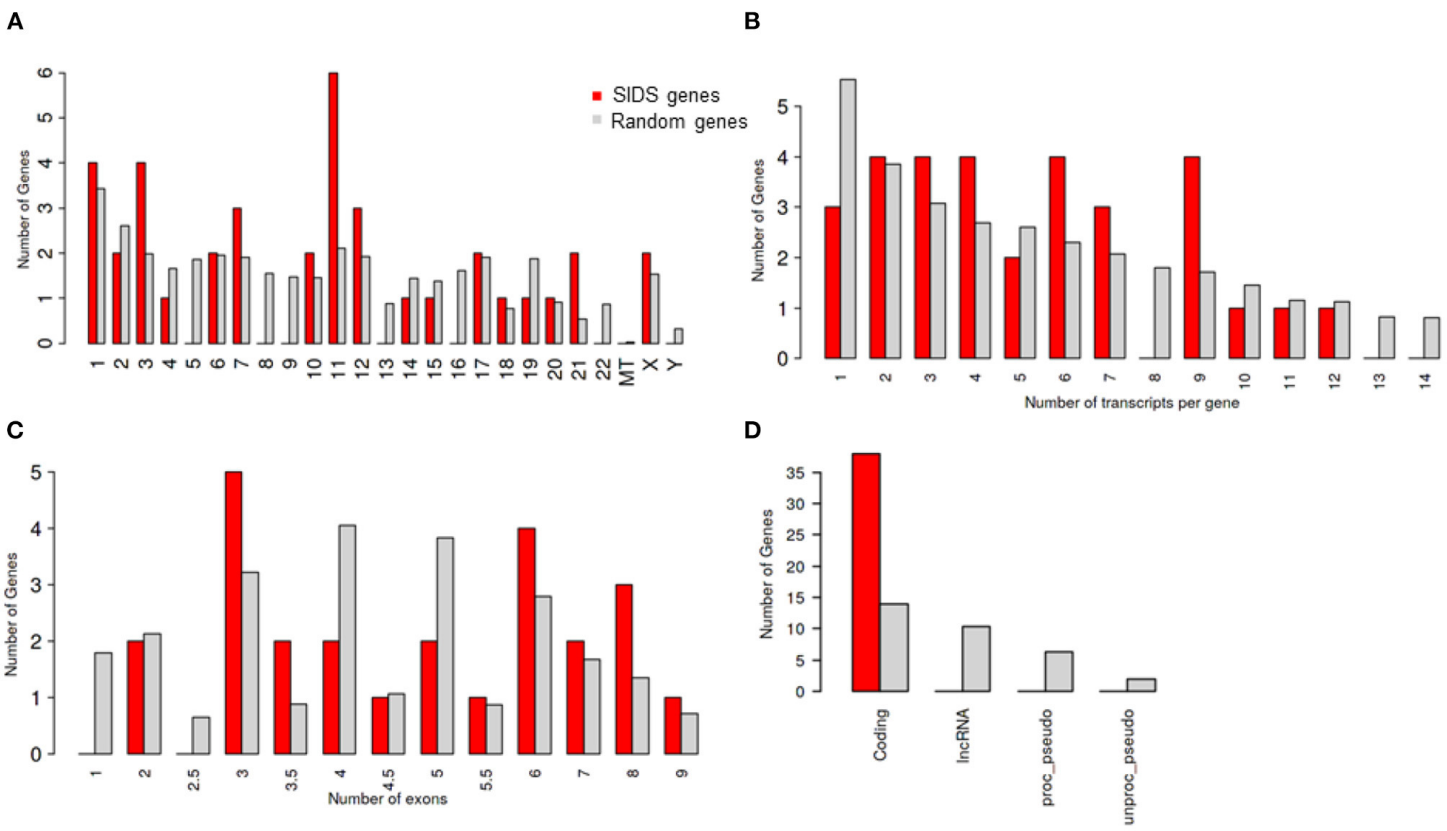
Genomic architecture of genes involves in SIDS **(A)** Distribution of SIDS genes on chromosomes Chi-squared test *P* = 0.43 **(B)** Number of transcripts isoforms per coding genes Chi-squared test *P* = 0.65 **(C)** Number of exons SIDS Chi-squared test *P* = 0.66 **(D)** Distribution of Gene type Chi-squared test *P* = 1.2 E-10.

The chromosome 11 harbored the maximum number of genes selected for the study, followed by chromosome 1 and 3 (Table 1; Figure 1A). The genes on chromosome 11 included the genes encoding for: potassium voltage-gated family channel (subfamilies Q and E), sodium channel beta subunits and potassium inwardly rectifying channel (subfamily J). The characteristics of SIDS genes are compared with the rest in the genome. Chi-square and Student's *t*-tests are run to see if the particular genes have special characteristics when compared with all the other genes in the human genome (Figures 1A–D). Figure 1B indicates that SIDS genes have more transcripts or isoforms, showing the complexity of the network. SIDS genes have been observed to contain more exons as compared to the random genes as shown in Figure 1C. Another interesting aspect observed through the computational analysis is that most of the SIDS genes are coding, which means that they code for proteins and involved in the regulatory mechanisms (Figure 1D).

**Table 1:** Genes extracted and investigated for association.

**No**.	**User ID**	**Ensembl Gene ID**	**Symbol**	**Gene type**	**Species**	**Chr**.	**Position (Mbp)**
1	CASQ2	ENSG00000118729	CASQ2	Protein_coding	Human	1	115.7000
2	NOS1AP	ENSG00000198929	NOS1AP	Protein_coding	Human	1	162.0697
3	TNNT2	ENSG00000118194	TNNT2	Protein_coding	Human	1	201.3590
4	RYR2	ENSG00000198626	RYR2	Protein_coding	Human	1	237.0422
5	DES	ENSG00000175084	DES	Protein_coding	Human	2	219.4184
6	CAV3	ENSG00000182533	CAV3	Protein_coding	Human	3	8.7338
7	GPD1L	ENSG00000152642	GPD1L	Protein_coding	Human	3	32.1057
8	SCN5A	ENSG00000183873	SCN5A	Protein_coding	Human	3	38.5481
9	SCN10A	ENSG00000185313	SCN10A	Protein_coding	Human	3	38.6968
10	ANK2	ENSG00000145362	ANK2	Protein_coding	Human	4	112.8181
11	DSP	ENSG00000096696	DSP	Protein_coding	Human	6	7.5416
12	TRDN	ENSG00000186439	TRDN	Protein_coding	Human	6	123.2163
13	CACNA2D1	ENSG00000153956	CACNA2D1	Protein_coding	Human	7	81.9464
14	AKAP9	ENSG00000127914	AKAP9	Protein_coding	Human	7	91.9409
15	KCNH2	ENSG00000055118	KCNH2	Protein_coding	Human	7	150.9450
16	CACNB2	ENSG00000165995	CACNB2	Protein_coding	Human	10	18.1407
17	BAG3	ENSG00000151929	BAG3	Protein_coding		10	119.6514
18	KCNQ1	ENSG00000053918	KCNQ1	Protein_coding	Human	11	2.4447
19	KCNE3	ENSG00000175538	KCNE3	Protein_coding	Human	11	74.4548
20	SCN4B	ENSG00000177098	SCN4B	Protein_coding	Human	11	118.1334
21	SCN2B	ENSG00000149575	SCN2B	Protein_coding	Human	11	118.1620
22	SCN3B	ENSG00000166257	SCN3B	Protein_coding	Human	11	123.6292
23	KCNJ5	ENSG00000129457	KCNJ5	Protein_coding	Human	11	128.8914
24	CACNA1C	ENSG00000151067	CACNA1C	Protein_coding	Human	12	1.9708
25	ALG10B	ENSG00000175548	ALG10B	Protein_coding	Human	12	38.3168
26	MYL2	ENSG00000111245	MYL2	Protein_coding	Human	12	110.9108
27	MYH7	ENSG00000092054	MYH7	Protein_coding	Human	14	23.4127
28	RANGRF	ENSG00000108961	RANGRF	Protein_coding	Human	17	8.2887
29	KCNJ2	ENSG00000123700	KCNJ2	Protein_coding	Human	17	70.1687
30	DSG2	ENSG00000046604	DSG2	Protein_coding	Human	18	31.4982
31	SCN1B	ENSG000000105711	SCN1B	Protein_coding	Human	19	35.0305
32	SNTA1	ENSG00000101400	SNTA1	Protein_coding	Human	20	33.4080
33	KCNE2	ENSG00000159197	KCNE2	Protein_coding	Human	21	34.3640
34	KCNE1	ENSG00000180509	KCNE1	Protein_coding	Human	21	34.4467
35	DMD	ENSG00000198947	DMD	Protein_coding	Human	X	31.0977
36	GLA	ENSG00000102393	GLA	Protein_coding	Human	X	101.3978
37	1-L	Not mapped	1-L	NA	NA	NA	NA
38	CACNA1	Not mapped	CACNA1	NA	NA	NA	NA
39	FBN1	Not mapped	FBN1	NA	NA	NA	NA
40	GPD	Not mapped	GPD	NA	NA	NA	NA
41	KCNE1L	Not mapped	KCNE1L	NA	NA	NA	NA

The potassium voltage-gated (K_v_) channels are the prototype members of membrane signaling proteins and they comprise the most complex, largest and evolutionary conserved family of ion channels (43). They are ubiquitous to all eukaryotic cells and are versatile in retaining membrane potential, balancing the cell volume and harmonizing the neural electric excitability (44). These channels allow millions of ions to selectively pass the membrane in a second and upon change in voltage or ligand concentration, their gates can open and close in milliseconds. The movement of solutes across the cell membrane produces a voltage difference that results in the generation of electric current. Homeostasis makes the fast-electrical signaling possible by maintaining the environment and content of the cell (increased concentration of sodium ions in extracellular fluid and blood and increased concentration of potassium ions inside the cytoplasm) (43). This homeostasis is maintained by the movement of ions across the membrane through the voltage gated channels. Humans contain 12 subfamilies of the K_v_ channels that comprise 40 genes encoding for the pore forming subunit of the channels. In the neurons innervating the muscle and cardiac muscle cells, they regulate the waveform of action potential (45).

The potassium voltage-gated channel subfamily Q member 1 (KCNQ1) encodes a voltage gated potassium channel. Two major functions of the KCNQ1 includes the repolarization of the cardiac tissue ensuing action potential and transport of water and salt in epithelial tissues (46). It contributes to diverse physiological processes by complexing with the subfamily E regulatory subunit 1 (KCNE1). There are five genes in the KCNE family that encode the regulatory subunit of transmembrane spanning ion channel. Their primary function is to complex with the K_v_ channels (47). Mutations in KCNQ1 and KCNE1 leads to malfunctioning of the channel and thus cause Romano-Ward syndrome and Jervell and Lange-Nielsen syndrome (48, 49).

In the present investigation, three sodium channel beta subunits genes were linked with SIDS. The Voltage gated sodium channels consist of a pore forming α subunit joined by two β subunits, thus forming a heterotrimeric complex that was first identified for their involvement in the upstroke of action potential. In mammals, the SCN (1-10) A genes encode the α subunit and SCN (1-4) B genes encode the β subunits. The sodium voltage-gated channel beta subunits (encoded by SCN4B, SCN2B and SCN3B genes) are not only the regulatory subunits of the transmembrane glycoprotein complexes but they also perform several functions independent of the α subunit and are involved in regulating cell excitability, affecting brain development and conferring distinct channel pharmacology (50). Defects in SCN4B, 2B and 3B genes (besides other problems) could result in different syndromes including SIDS (1, 51, 52).

A member of the potassium inwardly rectifying channel subfamily J was also found to be associated with SIDS—KCNJ5. This gene encodes the integral membrane protein of the inwardly rectifying potassium channels. Conditional to their type and location, these channels perform a variety of physiological functions. They permit the flow of potassium ions in rather out of cell and are regulated by G-proteins (53).

There were four genes on each of the chromosome 1 and 3 linked to SIDS. The genes on chromosome 1 included the Calsequestrin-2 (CASQ2), Nitric Oxide Synthase 1 Adaptor Protein (NOS1AP), Troponin T type 2 (TNNT2) and Ryanodine Receptor 2 (RYR2) genes.

The protein encoded by CASQ2 is a calcium binding protein that serves as a calcium depot for muscle function in the cardiac muscles. The precise control of calcium ions regulates the normal contraction and relaxation in the heart muscles which is essential for maintaining regular heart rhythm (54). If there occurs an alteration in calcium ions concentration then the gene TNNT2 encoded protein regulates the muscle contraction. Mutations in TNNT2 lead to dilated cardiomyopathy (enlarged heart effecting pumping) or familial hypertrophic cardiomyopathy (abnormal thickening of heart muscles leading to difficulty in pumping blood) (55, 56). The RYR2 gene encoded protein makes channels for the transport of calcium ions in the heart myocytes. These channels are embedded in the sarcoplasmic reticulum (outer membrane of cell structure) thus acting as storage depot for calcium ions. The RYR2 channels regulated the release of calcium ions (from the sarcoplasmic reticulum to the surrounding cells) in myocytes in response to the signals. Increase in calcium ion concentration leads to heart muscle contraction. The cardiac myocytes relax when the calcium ions are taken back to the sarcoplasmic reticulum (57).

The genes on chromosome 3 included the Caveolin-3 (CAV3), Glycerol-3-Phosphate Dehydrogenase 1 Like (GPD1L) and sodium voltage gated channels alpha subunits 5 and 10 (SCN5A and SCN10A) genes. CAV3 gene encodes the protein caveolin-3 that plays a role in muscle development along with the other two members of the family; CAV-1 and−2. CAV-3 is also involved in the signaling pathways and energy metabolism (58, 59). The GPD1L gene encodes a cytoplasmic protein associated with the cell membrane where it binds to the SCN5A. Mutations in the GPD1L has been reported to cause Brugada syndrome type 2 (BRS2) as well as SIDS (60). Mutations in CAV3 are responsible for the malfunctioning of SCN5A that also becomes a potential cause of SIDS (61).

### Gene Enrichment in Biological Processes

**Figure 2:**
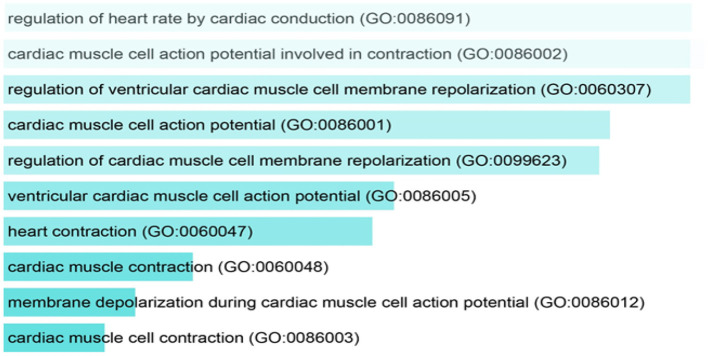
Pathway analysis showing the over represented SIDS genes in different biological pathways.

Gene enrichment analysis was performed to recognize the over represented genes in our data set that may have been associated with SIDS. The biological pathways that were enriched in certain genes were analyzed. The 40 genes undertaken in this study were found to be regulating different biological pathways (Table 2; Figure 2). Most of the genes were associated with cardiac related functions. Nearly 70% of the genes studied were involved in regulation of the heart rate by cardiac conduction and cardiac muscle cell action potential. About 80% of the genes were linked to cardiac contraction.

**Table 2:** Gene enrichment showing the number of genes associated to a particular biological process.

**Enrichment FDR**	**Genes in list**	**Total genes**	**Functional category**
1.5E-61	29	98	Regulation of heart rate
1.6E-60	27	70	Cardiac muscle cell action potential
2.5E-60	33	246	Regulation of heart contraction
9.5E-59	33	275	Heart contraction
2.3E-58	33	284	Heart process
6.6E-58	29	133	Cardiac muscle contraction
1.0E-57	33	299	Regulation of blood circulation
1.3E-57	28	111	Actin-mediated cell contraction
6.9E-55	28	136	Actin filament-based movement
7.2E-55	29	168	Striated muscle contraction
8.8E-53	23	52	Cardiac muscle cell action potential involved in contraction
1.1E-52	27	131	Action potential
3.1E-50	23	64	Cardiac muscle cell contraction
4.1E-50	31	353	Muscle contraction
3.5E-49	33	542	Blood circulation
5.8E-49	33	551	Circulatory system process
1.2E-47	33	603	Regulation of system process
3.3E-47	31	438	Muscle system process
8.9E-44	19	40	Regulation of heart rate by cardiac conduction
1.2E-43	24	145	Cardiac conduction
4.2E-42	29	450	Regulation of membrane potential
8.0E-40	24	205	Multicellular organismal signaling
3.8E-39	17	35	Ventricular cardiac muscle cell action potential
6.0E-39	28	485	Regulation of ion transmembrane transport
6.4E-39	26	336	Regulation of cation transmembrane transport
4.6E-37	28	566	Regulation of transmembrane transport
8.8E-37	26	405	Regulation of metal ion transport
1.9E-36	17	47	Membrane repolarization
5.5E-36	16	36	Regulation of membrane repolarization
1.4E-35	15	27	Ventricular cardiac muscle cell membrane repolarization

A normal heart contracts at regular intervals in a continuous fashion to supply oxygenated blood to the whole body and bring back the deoxygenated blood to the lungs (62). To maintain normal heart rhythm, a specialized tract of myocardial cell constitute the cardiac conduction system (63). The mechanical functioning of the heart depends on the production and propagation of action potential—electrical functioning. Between the two impulses, the heart contracts and relaxes followed by a period of refractoriness. This electrical functioning is manifested in the successive activation of cells in specialized regions of the heart—the pacemaker, and the propagation of action through the ventricles. The electrical activity of the myocardial cells is associated to the production of action potential in heart cells and its propagation along with the electromagnetic functioning of ventricles depends on the electrical coupling between cells mediated by gap junctions (64, 65). Activation and subsequent inactivation of ion channels that conduct depolarization (influx of Na^+^ and Ca^2+^) and repolarization (efflux of K^+^) generates myocardial action potential (66). The unidirectional propagation of excitation through the myocardium and normal cardiac rhythm generates due to the distinct waveforms of action potential in different regions of the heart that occurs due to the differences in the expression and/or properties of underlying ion channels (66–69). Inherited mutations in the genes encoding the ion channels or myocardial disease can lead to changes in the functional expression and properties of ion channels resulting in alterations in the action potential waveforms, and/or propagation thus predisposing the heart to life threatening arrhythmias (70). Structural anomalies in the conduction system can lead to lethal cardiac arrhythmias or heart block that might be linked to SIDS (71). Nearly 85 % of the sudden deaths are of cardiac origin—sudden cardiac death (72).

### Gene Regulation by Histone Modification

**Figure 3:**
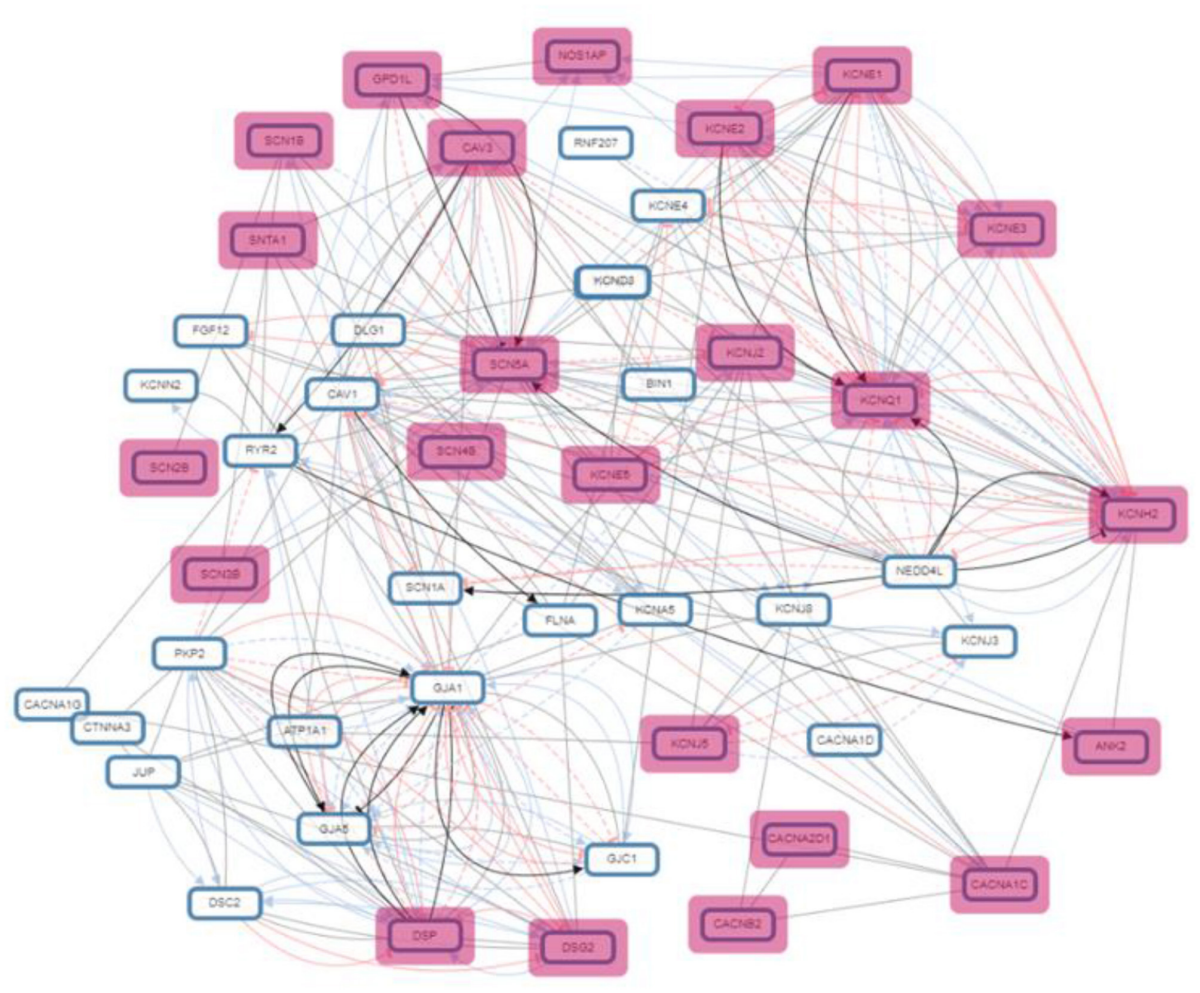
Pathway network illustrating the genes interaction in biological processes.

A ubiquitous and integral component of eukaryotic gene regulation is histone. The DNA is wrapped around histone octamers forming a structure called nucleosomes thus changing the accessibility and structure of DNA (73). Post-translational modifications in histones may occur by covalent addition of chemical groups (acetyl, methyl, phosphoryl, and small proteins) at specific residues (74), and occur frequently in the extruding tails of DNA that can change the nucleosome structure and also modulate gene expression by recruiting protein involved in gene regulation (73). The set of genes undertaken in the present study were found to be involved in four different epigenetic modifications: H3K27me3, H3K9me3, H3K4me3, and H3K36me3 (Figure 3; Table 3).

**Table 3:** Four different histone modifications found in present study.

**H3K27me3**	**H3K9me3**	**H3K4me3**	**H3K36me3**
KCNH2	DSP	CACNB2	DES
KCNJ5	RYR2	KCNE3	SCN5A
KCNE1	KCNJ5	CACNA2D1	GLA
SCN10A	CACNA2D1	NOS1AP	SCN1B
KCNE2	SCN5A	DMD	MYH7
KCNE3	SCN3B	CACNA1C	ALG10B
DMD	KCNJ2	SCN3B	CACNA1C
SCN5A	SCN1B	KCNJ2	CACNA2D1
SCN3B	SNTA1	DSP	KCNQ1
SCN4B	FBN1	DSG2	RANGRF
SCN1B	CACNB2	SCN1B	KCNE1
SCN2B	SCN10A	SNTA1	DSG2
DSP	SCN4B	KCNH2	ANK2
RYR2	SCN2B	SCN5A	TNNT2
DES	KCNE1	SCN4B	
BAG3	KCNQ1	KCNE1L	
KCNQ1	NOS1AP	ALG10B	
NOS1AP	MYH7	KCNQ1	
DSG2	KCNE3	RANGRF	
SNTA1	KCNE1L	FBN1	
FBN1	CASQ2	AKAP9	
CACNA2D1	GLA	RYR2	
ANK2	TRDN	GLA	
CACNB2	DMD		
KCNJ2	ALG10B		
MYH7			
CACNA1C			
GPD1L			
MYL2			
TNNT2			
KCNE1L			
AKAP9			
ALG10B			

Histone methylation is a type of epigenetic modification that allows alteration on chromatin structure without changing the underlying genomic sequence. They usually occur on lysine or arginine residue (75). H3K27me3 is a tri-methylation at the 27th lysine residue of the DNA packaging protein, Histone H3. This epigenetic modification forms heterochromatic regions thus downregulating the nearby genes (76).

The tri-methylated H3K27 signifies a highly conserved histone modification that is strongly linked to inactive gene promoters (77). This transcriptional silencing is produced and maintained by evolutionary-conserved histone-modifying enzymes—Polycomb Repressive Complexes (PRCs) (78). Along with H3K27me3, tri-methylation of histone 3 lysine 9 (H3K9me3) are the best-known histone modifications related to gene expression and heterochromatin. These modifications are carried out by enzymatic “writers” and are rectified by enzymes—“eraser,” to remove the modifications (79). H3K27me3 controls the expression of developmentally regulated genes (80, 81). H3K9me3 is abundantly found in human active gene promoters in several cell types and it represses the repetitive DNA elements and silencing factors encoding genes (79, 82).

Tri-methylation of histone 3 lysine 4 (H3K4me3) upregulates transcription by stabilizing the formation of pre-initiation complex (83). It binds to different protein folds that are present in chromatin remodeling and histone modifying factors, thus changes in levels of H3K4me3 can attribute to change in transcription (84). The tri-methylation of histone 3 at lysine 36 (H3K36me3) is firmly associated with actively transcribed genome regions and plays role in transcriptional activation. This modification is known to mark active genes. There is evidence to indicate a role of H3K36me3 in the DNA damage response, thus a reduced level of H3K36me3 could be linked with a decline in DNA repair efficiency (75).

Aberrant histone methylation plays a significant role in causing cancer and differentiation of stem cells. The transcriptional inhibition by H3K27me3 regulates the gene expression, affects chromatin structure, transcriptional activity of neighboring genes, availability of transcription factors, thus also normal cell development and disease progression (85, 86). In colorectal cancer patients, decreased levels of H3K27me3 lead to poor prognosis and resistance to chemotherapy (87). Also, loss of polycomb repression reduces H3K27me3 level, that leads to troublesome prognosis of several cancers. Mutations in the histone 3 drive tumorigenesis in pediatric high-grade gliomas (88). H3K27me3 and H3K9me3 may also play a role in colorectal cancer due to alteration in expression level (89).

As a result of excess and abnormal methylation reactions, cancer cells require a higher amount of methionine and thus become methionine addicted (90–92). This addiction prevents cancer cells from proliferation under methionine restriction. Histone methylation status of H3K9me3 and H3K4me3 were volatile in cancer cells and their levels were drastically dropped be methionine restriction in cancer cells (93).

### Role of Transcription Factors in Genes Involved in SIDS

**Figure 4:**
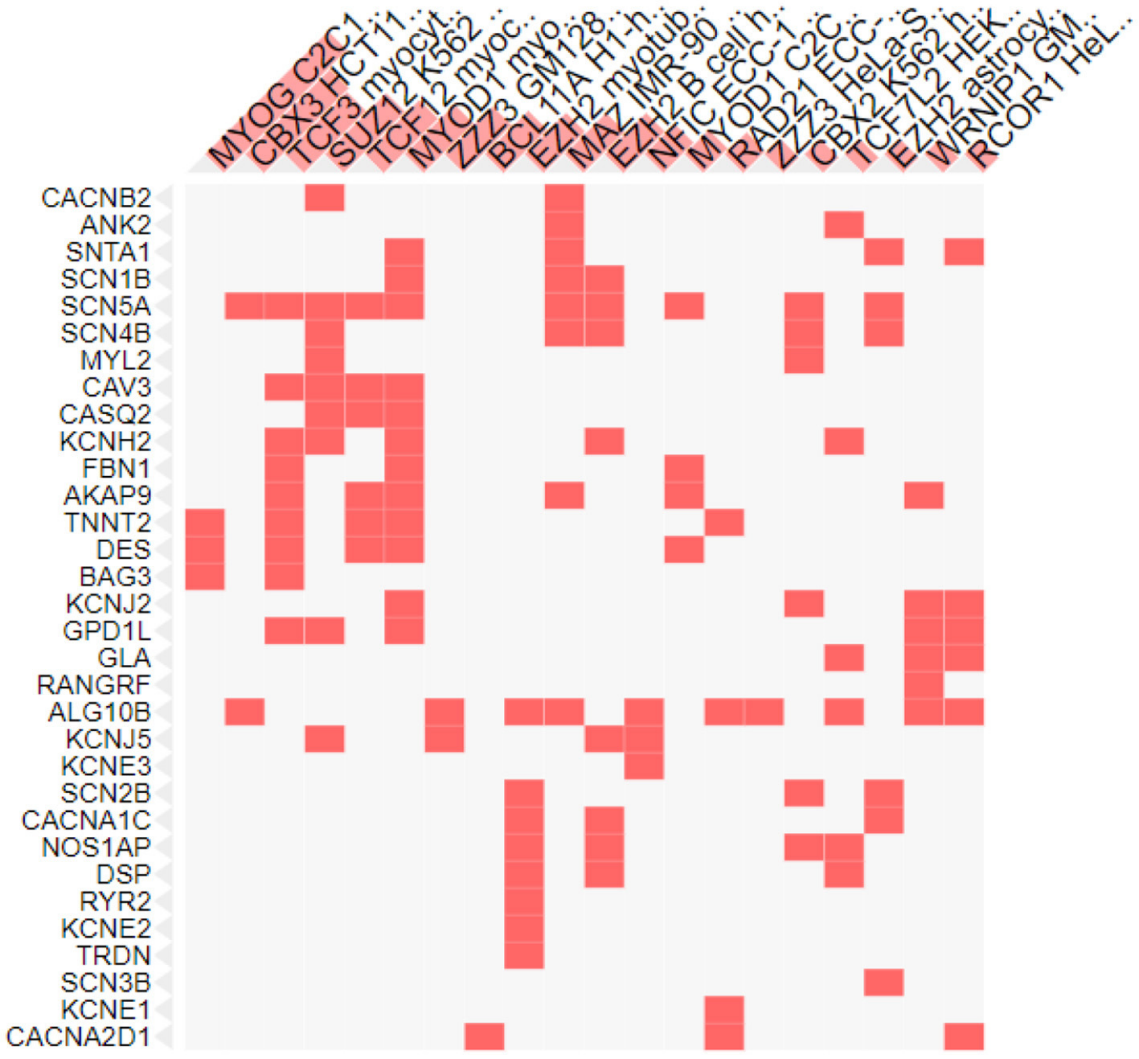
Transcription factors associated with SIDS genes. X-axis shows the transcription factors and y axis shows the genes under study.

Transcription factors are the proteins that regulate the transcription rate by interacting with the regulatory DNA sequences lying 5' upstream to the target genes. There are different families of transcription factors including the helix-turn-helix, helix-loop-helix, zinc finger, beta sheet motifs and basic protein-leucine zipper (94). Different transcription factors are correlated with the genes involved in SIDS (Figure 4). The maximum number of genes (12 genes) were found to be linked with the Myogenic differentiation 1 (MyoD1) transcription factor that promotes the expression of muscle-specific genes and has role in muscle differentiation along with myogenic factor 5 (MYF5) and Myogenin (MYOG) (95). The MYOD1 plays a crucial role in accelerating the transcription of p21 (a cyclin dependent kinase that acts as a tumor suppressor) and myogenin to remove the cells from cell cycle and terminate multiplication of differentiated myocytes (96). Functional dysregulation and abnormal expression of MyoD1 has been reported in retinoblastoma (97), head and neck cancer (98), breast cancer (99) and lung adenocarcinoma (100). The MyoD1 was also significantly under expressed in gastric cancer (101).

Nine genes were also associated with the Transcription factor 3 (TCF3), encoded by the TCF3 gene and belongs to the helix-loop-helix family [E protein (class 1)] of transcription factors. These proteins are essential for the B and T lymphocyte development, thus play a crucial role in lymphopoiesis. Deletion or under expression of this gene is linked with lymphoid malignancies including pre-B-cell acute lymphoblastic leukemia and childhood leukemia (102). TCFs was recognized as the key regulator in the wilms tumor (pediatric kidney tumor) (103) and was found to act as a promoter in cervical cancer and associated with the development of cervical squamous cell carcinoma (104).

The lowest number of genes were found to be linked to the MYOG and chromobox protein. Three of the genes were associated with the muscle-specific transcription factor MYOG (Figure 4) essential for skeletal muscle development. It is a member of the Myogenic Regulatory Factor family of transcription factors that are main controllers of vertebrate muscle gene expression at early and adult myogenesis (105, 106). Severe muscle deficiencies were found in MYOG gene knockout mouse (107). Any abnormality in Myog transcription factor can lead to defects in regulation of muscle homeostasis, muscle growth and development, and reduced myofiber growth (108).

Two genes under study were associated with a transcription factor from the heterochromatin protein 1 family member, Chromobox protein homolog 3 (CBX3), that in involved in a variety of cellular processes like: DNA repair, gene regulation (by transcriptional activation or repression), cell differentiation and growth and epigenetic modification (109–114). CBX3 is upregulated in many cancers and plays an important role in the progression of tumors. The expression of CBX3 was elevated in human colorectal cancer (115), prostate cancer (116), breast cancer (117) and pancreatic cancer (118). In breast and lung cancer over expression of CBX3 leads to poor prognosis (117, 119).

### The Key Regulator of Putative SIDS Genes—“mir-133a-3p”

MicroRNA (miRNA) are a set of highly conserved, endogenous, small non-coding RNAs molecules that range in size from 18 to 23 nucleotides and supress the gene expression by interacting with 3' untranslated regions (UTR) of target mRNAs (120). The miRNAs are significant in carcinogenesis and a number of evidences support that by adapting to the pathophysiological progressions, they may act as oncogenes or tumor suppressor genes (121–124). miRNAs are predicted as a tool for early diagnosis and treatment of cancer patients to extend their survival time (125–128).

**Figure 5:**
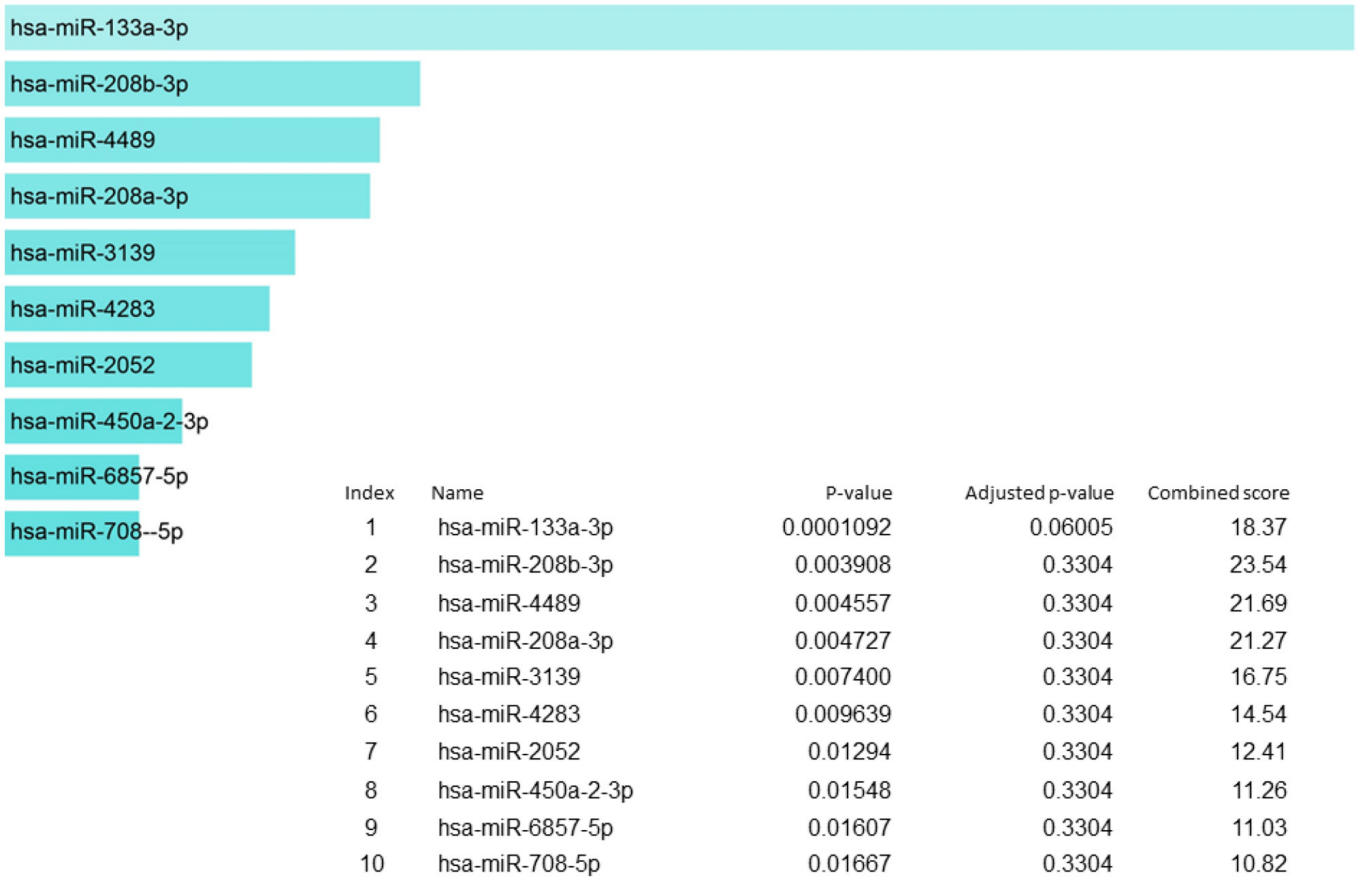
miRNA regulation of SIDS genes showing the key regulator miR-133a-3p at the highest position.

In our candidate set of SIDS genes, miR-133a-3p is the highest reported (Figure 5). MiR-133-3p is a member of the miR-133 family that has two copies, miR-133a-1 and miR-133a-2, in humans. miR-133a-3p has multifaceted functions such as controlling proliferation and differentiation of myoblast (129), constraining proliferation of embryonic cardiomyocytes (130) and eluding genetic cardiac hypertrophy (131). In several human malignancies, miR-133a-3p is one of the recurrently downregulated miRNA which proposes that it may have a crucial role in tumor development in numerous malignancies, including non-small cell lung cancer (132), colorectal cancer (133), breast cancer (134), ovarian cancer (135), bladder cancer (136) and prostate cancer (137). The miR-208b-3p was the second in line in our set of genes but it was not regulating to the extent as miR-133-3p does. The miR-208b is among those miRNA species that are outstanding candidate as biomarkers for the diagnosis and prognosis of different diseases owing to their remarkable stability and detectability in peripheral blood or plasma, since the levels of these circulating miRNAs are symptomatically altered in individuals in several diseases (138–141). For the detection of cardiac diseases, cardiac specific miR-208b-3p have been utilized as promising biomarkers (142).

## Discussion

SIDS is multifactorial and its cause remains undiagnosed so far. Genetic factors are least known and least explored. SIDS may be associated with variants in various genes. This study highlights the fact that genetic studies related to SIDS are scarce and there is insufficient literature reported so far. As there is genetic diversity due to genetic polymorphisms in different populations and also within same populations, so there is a dire need to explore SIDS on these lines.

A recent study from Germany (143) evaluated 31 SIDS cases via Next Generation Sequencing and reported the facts similar to our findings that cardiac channelopathy and cardiac myopathy genes should be investigated to find a link with SIDS. They used the pulmonary tissue samples after autopsy. We have also found the genes involved in cardiac channelopathy and cardiac myopathy to be associated with SIDS in various studies. In approximately 30% cases of SIDS, cardiac channelopathy linked genes were observed to be a monogenic cause of deaths (144). In 3.5% of the SIDS cases, a genetic polymorphism in a cardiac myopathy gene was reported (145). Although it is very important to detect and evaluate the genetic variants in order to counsel the family in a SIDS case, but it should be evaluated and labeled carefully as “pathogenic” or “informative” as 4.3% was found to be pathogenic and 13% as informative in the recent study mentioned above (143) which is much lower that assumed previously.

Various case reports have identified genetic predisposition in previously healthy infants who became victims of SIDS later on. In one report, SIDS with dysgenesis of the Testes syndrome (SIDDT), which is a rare condition caused by TSPYL1 gene, was reported for the first time in 2004. It is characterized by sudden cardiac or respiratory arrest, disordered testicular development, neurologic dysfunction. exome sequencing revealed homozygosity for a frameshift variant in TSPYL1 (c.725_726delTG, p.Val242GlufsTer52) consistent with a diagnosis of SIDDT, explaining many of the clinical features (146). Cerebellum has also been reported in a recent case series of four infant deaths and upon investigation, cerebellar heterotopia of infancy was found which is a distinct malformation of the cerebellum. It should be further explored (147). Exome sequencing was done in 10 SIDS cases and SCN1A was identified in two victims with hippocampal abnormalities. One had SCN1A G682V and other had 2 SCN1A variants in cis: L1296M and E1308D variants which were previously known to be associated with epilepsy (148).

In a rare case report of 15 day old infant who died unexpectedly and suddenly, whole exome sequencing was performed and two novel mutations in the *CLCKB* gene were found and lead to a molecular diagnosis of Bartter's syndrome Type III. This case highlights the importance of whole genome sequencing in such cases for proper diagnosis (149). In another case report, a 3 month old infant was found dead while sleeping and postmortem CT scan showed fatty attenuation in the liver parenchyma. Matrix assisted laser desorption/ionization imaging mass spectrometry (MALDI–IMS) analysis was conducted to further investigate the underlying mechanism of lipid accumulation. This revealed a significant accumulation of C14:1 acylcarnitine in the liver, proposing a very long-chain acyl-CoA dehydrogenase (VLCAD) deficiency. Genetic analysis showed two novel mutations in ACADVL gene (150).

In a recent study, brain, heart, liver and kidney weights of SIDS victims were compared to controls. There was a large samples size of 291 SIDS and 294 controls obtained from Australia and Russia. Brain, thymus, liver and body weights were significantly higher among SIDS as compared to controls. Heart weight was significantly less in SIDS cases. Major difference was observed among thymus and brain weights and sizes. Heavier thymus points out to a possible infectious link to SIDS demises and larger brain sizes and weights shows a possible neuropathological roles (151). Larger thymus highlights the possible involvement of immunological genes which may be explored in this context. Genes related to neurotransmitters, their receptors and cardiopulmonary system should also be investigated further. Larger livers show a possible role of biochemical parameters and fatty acids (152) and need to be studied in detail. In another study, the frequency of extramedullary haematopoiesis (EMH) was observed to be significantly higher in SIDS victims as compared to controls which may be due to anemia associated with intrauterine hypoxia or infections (153).

Heterozygous gene mutation in PHOX2B, was reported in another case report of 28 day old female infant who died while lying on her bed in prone position. There were no pathological findings in postmortem investigation. PHOX2B gene is associated nervous system development and responsible for breathing control. Any mutation in this gene may lead to congenital central hypoventilation syndrome (154). Neonatal hypertrophic cardiomyopathy is a genetic disorder caused by mutations in MYBPC3 gene (p.Glu258Lys and IVS25-1G>A). It is associated with cardiac myopathy in neonates and leading cause of mortality and morbidity (155). In another case report, a sudden death of a 1 month old male infant while asleep was reported who was apparently healthy before the demise. This case was attributed to a combination of unavoidable events of congenital cardiac alterations, neuropathological alterations in brainstem and mutation in transporter gene (156). A case report on SIDS victim two female infants who were sisters as well, revealed a homozygous L2HGDH gene mutation. This gene is known to be associated with inherited metabolic disorders if mutated but it was first time reported in SIDS victims in this report published in 2009 (157).

Twin pregnancy and birth is also a risk factor for increased complications and SIDS. Koehler et al., published a review in 2001 on SIDS and Simultaneous Sudden Infant Death Syndrome (SSIDS) cases based on worldwide review of literature published from 1991 to 1998. SSIDS cases included the death of a pair of twins occurring suddenly without known reason. 41 SSIDS cases were found in this given time duration and they proposed a three point criteria for the cases to be considered as SSIDS and these included the location of death, a summary of the circumstances surrounding the deaths and the evaluation of these cases in terms of a proposed definition of SSIDS. According to this criteria, only 12 pairs of twins met all the three criteria and no conclusions were drawn (158). In a case report published in 2013, both the 10 weeks old twin infants died while sleeping on their backs. They were healthy and well before the sad event. Post-mortem investigations revealed petechial hemorrhages on the visceral pleura, epicardial surface and thymus gland. Histological findings showed pulmonary edema, intra-alveolar hemorrhage and minor lymphocytic infiltration in both infants, indicative of infection (159).

SIDS was significantly more common in monozygotic twins and of same gender during a retrospective study in England and Wales from 1993 to 2003 conducted by the office of National Statistics (160). In another study with conflicting results from United States, 23,464 singleton SIDS and 1,056 twin deaths were reported during 1987-1991. It was concluded that twins do not appear to be an increased risk factor for SIDS, twins dying of SIDS is uncommon while twins dying on same day is extremely rare incident (161). In another case report, a sudden infant death occurred in a 22 month old male twin infant while the other remained healthy. They contracted gastroenteritis and were given an antibiotic containing pivalic acid which is sometimes causing hypocarnitinemia. After post-mortem investigations and later on the genetic investigations, it was revealed that the fatal incident happened due to the deficiency of fatty acid oxidation accelerated by an antibiotic containing pivalic acid and the virus infection in this SIDS victim was containing the the thermolabile variant of carnitine palmitoyl transferase 2 (CPT2) gene. All factors alone are not fatal but cause SIDS when come in combination (162).

A case of sudden death of 1 month old male infant was reported by Donatella Mecchia et al. (156), attributing the death to cardiac, nervous system and genetic involvement. Polymorphism in serotonin transporter gene 5-HTT was observed (156). In another case report, a genetic analysis of 104 sudden cardiac death related genes was performed through Next Generation Sequencing (NGS). NGS identified 7 variants, 2 variations in genes *AKAP9, EN1* were known previously as pathogenic, four variations in genes *KCNE3, PKP2, VCL*, and *TTN* had no clinical significance known, 7th variation in gene *TTN* was novel (163). In a molecular autopsy study of 50 cases of sudden young death <45 years old, during a period of 2 years was performed by exome sequencing. For exome, variants in *TTN* gene were the most abundant, having 56 variants, followed by *MUC16* having 53 variants and *SSPO* having 49 variants. *TTN* is the gene encoding titin which is the largest protein of the human genome having 305 kbs and is involved in cardiomyopathies. *MUC16* or mucin 16 is a 132 kb gene coding for mucin protein which is a glycoprotein involved in cancers and SSPO (subcommissural organ spondin) is a 58 kb gene that codes for a protein involved in neuronal aggregation. All the above cases had negative autopsy findings but molecular autopsy revealed these genetic variations (164). Although these cases were not SIDS but it must be explored in SIDS as well and similar strategy may by used for molecular autopsy of SIDS.

In a whole exome sequencing study of 161 SIDS cases from Europe, 192 genes associated with cardiovascular and metabolic diseases were investigated. Potentially causative genes were identified in 20% of the SIDS cases, 9% of the genes were associated with channelopathies, 7% with cardiomyopathies, 1% with metabolic disorders (144). A molecular study conducted on 41 cases of sudden unexpected death in infancy, analyzed 86 sudden cardiac death related genes. 63 variants in 35 cases were validated. Most of these variants were located on cardiomyopathy genes or channelopathy genes (165). NGS was performed as a molecular autopsy tool for 16 cases of sudden unexpected deaths in youngs, <35 years old. Twenty three genes associated with inherited cardiac channelopathies were screened. An average 200 variants per case were identified and after correlating them with the clinical data and molecular findings, 4 likely pathogenic variants were identified in three cases in the genes *KCNH2, ANK2, SCN5A, and RYR2* (166). In a molecular autopsy on 141 SIDS cases, <6 months of age and 133 non/infants (19-58 years old) six major channelpathy genes, *KCNQ1, KCNH2, SCN5A, KCNE1, KCNE2, and RyR2* were characterize in 274 cases which were autopsy negative. 22 previously known variants linked to cardiac channelopathy and 24 novel variants were identified among infants (13.5%) and non-infants (19.5%). Most of these variants involved *SCN5A* gene (68.4% in infants and 50% in non-infants) which is a sodium channel alpha subunit. This study highlighted the role of cardiac channel genes in sudden deaths of infants and non-infants (167).

In our previous studies, including post-mortem studies, *in silico* and *in vitro* studies, we have proposed the involvement of Substance P/Neurokinin 1 Receptor and the gene encoding SP, Tachykinin 1 *(Tac 1)* to be involved in SIDS (168–172). We emphasize further studies to be conducted on this pathway as it is involved in the breathing control mechanism and a genetic mutation in its gene can be deleterious. We have also proposed the involvement of neuropathology in COVID-19 as well and proposed the same SP/ NK-1R mechanism which contributes in the neuropathology of SIDS. The same phenomenon may also be involved for lesser implications in children and infants as compared to the adults (173). Current study also presents the involvement of these candidate genes in other biological processes. We have also highlighted the importance of gene expression and its association with SIDS by discovering the histone methylations, gene enrichment pathways, transcription factors and micro RNAs reported so far. Gene regulation has utmost importance in these cases and must be investigated further. This study provides a comprehensive analysis and presents the genetic determinants which need further investigation. It will pave way for other researchers who are trying to find out the genetic causes of SIDS. Next Generation sequencing may be an important tool in this regard.

## Conclusions

A number of genes mainly potassium and sodium channel genes may be associated with the devastating phenomenon of SIDS. Future studies should be conducted to explore the properties of these genes in relation to SIDS so that vulnerable infants and neonates may be diagnosed timely before the sad demise. Preventive measures can be adopted to avoid such incidences. Next generation sequencing and careful interpretation of the gene variants is required to reveal these facts.

## Data Availability Statement

The datasets presented in this study can be found in online repositories. The names of the repository/repositories and accession number(s) can be found in the article/supplementary material.

## Author Contributions

RM and SAS planned, conceived, and designed and supervised the study. MK, MA, SAG, and SK did data extraction and write-up. HN, AM, HM, SH, and AH worked on revisions, write-up, and analysis. MA and AB did analysis, write-up, and critical review. All the authors have contributed to the article and approved the submitted version.

## Conflict of Interest

The authors declare that the research was conducted in the absence of any commercial or financial relationships that could be construed as a potential conflict of interest.

## Publisher's Note

All claims expressed in this article are solely those of the authors and do not necessarily represent those of their affiliated organizations, or those of the publisher, the editors and the reviewers. Any product that may be evaluated in this article, or claim that may be made by its manufacturer, is not guaranteed or endorsed by the publisher.
